# P-733. Incidence Rates of Anogenital Warts in Adult Men and Women

**DOI:** 10.1093/ofid/ofaf695.944

**Published:** 2026-01-11

**Authors:** Marian Awad, Stefano Valente, Dorothy A Machalek, Xuedan You

**Affiliations:** Eisai Inc, Piscataway, NJ; MSD Italy, Rome, Lazio, Italy; University of New South Wales, Melbourne, Victoria, Australia; Merck & Co., Inc.,, Rahway, NJ

## Abstract

**Background:**

Anogenital warts (AGW) are a common manifestation of human papillomavirus (HPV) infection in men and women. Approximately 90% of cases of AGW are caused by HPV types 6 and 11. Prophylactic HPV vaccines have high efficacy in preventing infection with these types. As HPV vaccination programs expand, updated data are needed to assess the ongoing global AGW burden across the lifespan. This review is aimed at examining the age specific trends in the incidence of AGW by sex.

**Figure:**
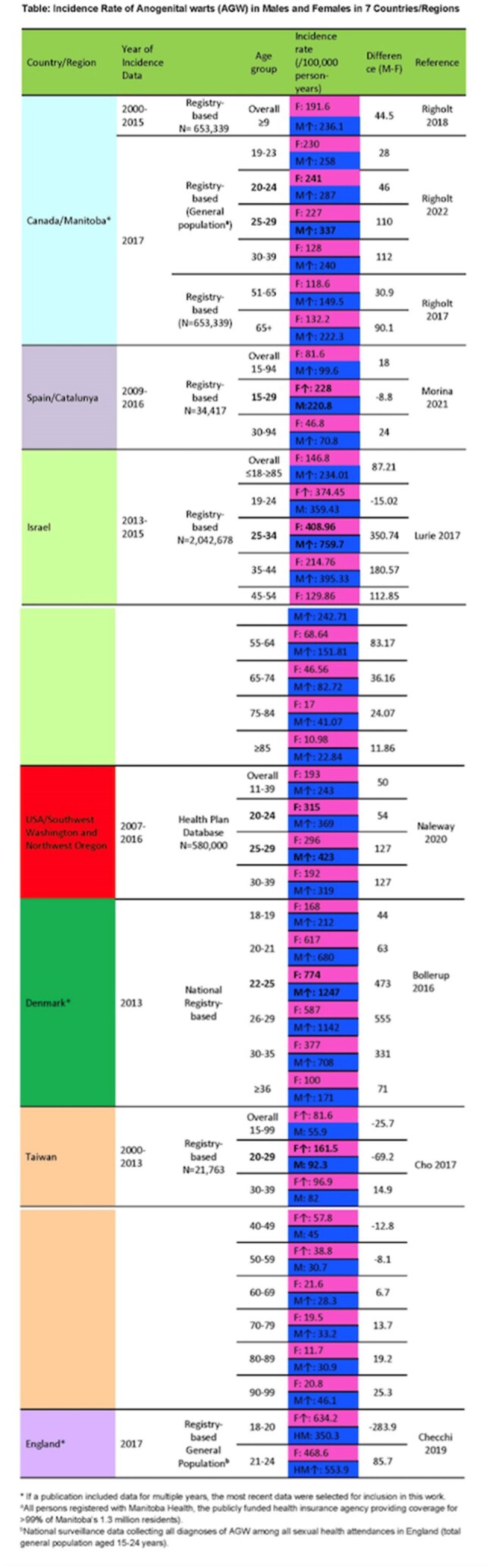

**Methods:**

This analysis includes studies that reported age-stratified AGW incidence rates in adults (18+) from a larger global systematic literature review (PROSPERO ID: CRD42023489504). Publications were retrieved from Embase, MEDLINE, and Evidence-Based Medicine Reviews (01/2013–10/2023) and conference proceedings (01/2021–12/2023).

**Results:**

Nine studies from 7 countries/regions were included. Five countries/regions reported overall incidence and 4 displayed men having higher incidence than women (99.6-243/100,000 person-years (py) vs 81.6-193/100,000 py). The difference between sex ranged from 18 in Catalunya, Spain to 87.2 in Israel per 100,000 py. Five studies reported incidence in more than two age groups. The highest incidence was reported in men and women aged 20-35. In Canada, Israel, USA, and Denmark men reported higher incidence in all age groups, except in 19-24 years in Israel. There was a decline in incidence with increasing age for both sexes, but the decline was less pronounced in men than women. In women incidence generally declined after age 25-30, and in males it continued to be high at ages 30-35 in Denmark (708/100,000 py vs 377/100,000 py), 30-39 in Canada and USA (240-319/100,000 py vs 128-192/100,000 py) and 45-54 in Israel (243/100,000 py vs 130/100,000 py). The same findings were not observed in Taiwan.

**Conclusion:**

The analysis revealed that men display higher incidence than women, especially at older ages. This data highlights the disease burden faced by men, further supporting the need to include males in HPV immunization programs.

**Disclosures:**

Dorothy A. Machalek, PhD, MSD: Honoraria|MSD: Travel Xuedan You, PhD, Merck & Co., Inc., Rahway, NJ, USA: Employee

